# Evaluation of Chemical Interactions between Small Molecules in the Gas Phase Using Chemical Force Microscopy

**DOI:** 10.3390/s151229823

**Published:** 2015-12-04

**Authors:** Jieun Lee, Soomi Ju, In Tae Kim, Sun-Hwa Jung, Sun-Joon Min, Chulki Kim, Sang Jun Sim, Sang Kyung Kim

**Affiliations:** 1 Center for Biomicrosystems, Korea Institute of Science and Technology (KIST), Hwarangno 14-gil 5, Seongbuk-gu, Seoul 136-791, Korea; 114013@kist.re.kr (J.L.); ssumi0115@gmail.com (S.J.); fresh1606@naver.com (I.T.K.); 2 Department of Chemical and Biological Engineering, Korea University, 5-ga Annam-dong, Seongbuk-gu, Seoul 136-713, Korea; simsj@korea.ac.kr; 3 Center for Neuro-Medicine, Brain Science Institute, Korea Institute of Science and Technology(KIST), Hwarangno 14-gil 5, Seongbuk-gu, Seoul 136-791, Korea; shjung8748@gmail.com; 4 Department of Applied Chemistry, Hanyang University, Sangnok-gu, Ansan, Gyeonggi-do 15588, Korea; sjmin@hanyang.ac.kr; 5 Center for Sensor System Research, Korea Institute of Science and Technology (KIST), Hwarangno 14-gil 5, Seongbuk-gu, Seoul 136-791, Korea; chulki.kim@kist.re.kr

**Keywords:** chemical force microscopy, micro-contact printing, lateral force image, benzene specific peptide

## Abstract

Chemical force microscopy analyzes the interactions between various chemical/biochemical moieties *in situ*. In this work we examined force-distance curves and lateral force to measure the interaction between modified AFM tips and differently functionalized molecular monolayers. Especially for the measurements in gas phase, we investigated the effect of humidity on the analysis of force-distance curves and the images in lateral force mode. Flat chemical patterns composed of different functional groups were made through micro-contact printing and lateral force mode provided more resolved analysis of the chemical patterns. From the images of 1-octadecanethiol/11-mercapto-1-undecanoic acid patterns, the amine group functionalized tip brought out higher contrast of the patterns than an intact silicon nitride tip owing to the additional chemical interaction between carboxyl and amine groups. For more complex chemical interactions, relative chemical affinities toward specific peptides were assessed on the pattern of 1-octadecanethiol/phenyl-terminated alkanethiol. The lateral image of chemical force microscopy reflected specific preference of a peptide to phenyl group as well as the hydrophobic interaction.

## 1. Introduction

Chemical force microscopy (CFM), derived from atomic force microscopy (AFM), works under atmospheric or aqueous environments with high spatial resolution and force sensitivity. This powerful tool measures inter- and intra-molecular forces between chemicals on a cantilever tip and molecular layer on a substrate. CFM has been used to evaluate the interactions between biomolecules such as complementary oligonucleotides, antibody/antigen and receptor/ligands *in situ* [1,2,3]. Furthermore, specific receptors of live cells have been imaged under physiological conditions by modifying tips with the relevant ligands [4,5]. The mechanism of biofilm formation was inferred from the interaction between biomolecules and hard surfaces. Bacterial particles have shown different affinities to various surfaces depending on their topology and chemical properties [6,7].

In contrast to biomolecules in aqueous conditions, small molecules under atmospheric conditions have not been closely investigated. To date, hydrophilic and hydrophobic chemicals have been analyzed to characterize physical forces such as electrostatic or van der Waals effects. This chemical force in the gas phase is known to be weaker than the binding forces of biomolecules in the aqueous phase. However, atmospheric conditions are advantageous for measurements of weak interactions due to the absence of damping media. Measurements of chemical forces in the gas phase are important for evaluating specific molecules recognizing certain gas targets. For example, those measurements are applicable to an increasing number of scientific studies reporting various types of recognizing molecules such as oligonucleotides, oligopeptides, and protein receptors that interact selectively toward gaseous organic molecules [8,9,10,11,12]. Yet, those specific receptors have not been investigated systematically due to the limited measures of molecular interactions in gas phase.

Comparison of interactions between receptors and target in the gas phase is still challenging due to weak binding between the molecules. These specific interactions have been analyzed by Fourier transform infrared spectroscopy (FTIR) and AFM [13,14]. FTIR can detect molecular interactions between the recognition layer and gas vapor. Meanwhile, water molecules are a major interfering agent in spectroscopy such that water vapor has to be removed carefully during measurements. When CFM is used under atmospheric conditions, the interaction between the tip and sample substrate is also sensitive to environmental humidity [15,16,17,18]. Thus, careful control over humidity is required for reliable and consistent measurements. 

**Scheme 1:**
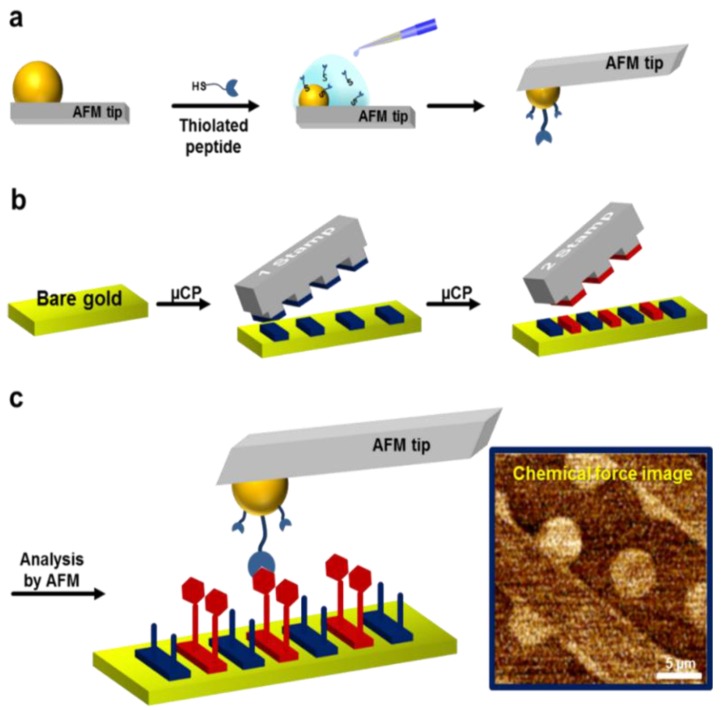
The schematic presentation of the strategy for fabricating a mixed organic pattern and analysis by AFM. (**a**) Preparation of gold ball tip modification using thiolated peptide. (**b**) Chemical was printed on a bare gold surface and the second stamp was printed the surface sequentially. (**c**) The chemical pattern was analyzed by AFM.

In this paper, we integrated a gas control system (Supplementary Information, Figure S1) and measured force-distant curves and lateral force to evaluate the interaction between chemically-modified AFM tips and differently functionalized organic molecular monolayers, as shown in Scheme 1. For the case of the derivatized form of gas targets, the results supported a direct comparison of the affinities between targets and receptors in gas phase. To fabricate a surface composed of organic molecules, alkanethiols, are are known to self-organize into well-ordered and densely packed films [19,20], were used as the organic molecular layer.

## 2. Materials and Methods

### 2.1. Materials

11-Mercapto-1-undecanoic acid (MUA), 6-mercapto-1-hexanol (MCH), 1-undecanethiol (UDT), 11-amino-1-undecanethiol (AUT), and 1-octadecanethiol (ODT) were purchased from Sigma Aldrich (St Louis, MO, USA). Ethanol and acetone were obtained from Junsei chemical Co., Ltd. (Tokyo, Japan). Polydimethylsiloxane (PDMS) was purchased from Dowhitech Silicone Co., Ltd. (Goyang-si, Korea). Phenyl terminated alkanethiol (PTA) was synthesized as described is Figure S2 (Supplementary Information). Thiolated benzene-specific peptides were prepared by phage-display library screening [21]. The peptides were purchased from KAAL (Seoul, Korea).

### 2.2. Preparation of Organic Monolayers

PDMS stamps were fabricated by SU-8 photolithography and the PDMS replica process (Supplementary Information, Figure S3-A–E) [22,23,24]. The PDMS stamp was immersed in 1 mmol/L of MUA in ethanol for 10 min. Excess MUA solution on the stamp was removed under a stream of N_2_. The MUA-coated stamp was printed on gold surfaces (1 × 1 cm) for 1 min (Figure S3-G). Then, MUA printed surfaces (rectangular pattern, 5 µm wide) were immersed in 1 mM of UDT or MCH in ethanol for 24 h. The resulting surfaces were washed with 99.9% ethanol and dried under a stream of N_2_. Next, we prepared a mixed patterned surface of MUA/ODT. MUA and ODT were first dissolved in ethanol at concentration of 1 mmol/L. The fabricated PDMS stamp was immersed in MUA or ODT solution and kept at ambient temperature for 10 min, and then the excess MUA or ODT solution on the stamps was removed by N_2_. The patterns of the two stamps were 4 µm lines for MUA and 5 µm squares for ODT. The wet stamps were printed on gold surfaces for 1 min. Then, we prepared a mixed patterned surface of PTA (line, 4 µm wide)/ODT (circle, 5 µm in diameter) by the same method described above (Figure S3-H).

### 2.3. Immobilization of Peptide and Amine Groups on Au-Coated Ball Ttips

In order to modify AFM probes with well-defined chemical functionalities, SiO_2_ ball tips (1 µm in diameter; Novascan, Ames, IA, USA) were coated with a thin layer of gold. Solution of thiolated probes (1 mmol/L) as peptides (benzene specific peptide sequence: N terminus-AAGDMMAPDPAC-C terminus, random peptide sequence: N terminus-AVPSGQAEAD PAC-C terminus) and 1 mmol/L solution of AUT were loaded to gold coated tips for 2 h in ethanol individually. The modified tip was rinsed in ethanol and dried under N_2_.

### 2.4. Environmental Controlled System for Force Measurement in the Gas Phase

Since the interaction of a self-assembled monolayer (SAM) is influenced by environmental conditions such as relative humidity, temperature and pressure, an environmentally controlled system is necessary. For this purpose, we built equipment for controlling humidity and gas (Figure S1-A,B) that consisted of: (1) a closed chamber; (2) nitrogen gas lines for purging and gas bubbling; (3) one volume flow meter (0–1 L/min.; Cole-Parmer, Inc., Vernon Hills, IL, USA) that was used to control flow rate; (4) a homemade bubble generator to increase humidity; and (5) a dehumidifier (Drierite, Xenia, OH, USA) to reduce humidity levels in the chamber. As shown in Figure S1-C, two operations were performed for nitrogen purging and water vapor addition. The gas was controlled with valves switching the flow paths of gases. By flowing nitrogen gas through the bubble generator with de-ionized water, the humidity could be increased and maintained over 85% relative humidity (RH). Using this system, we could fix a constant humidity for each measurement ranging from 10% to 85% RH.

### 2.5. AFM Instruments and Measurement

AFM measurements were performed using two commercial instruments, a Nanoscope IIIa (Digital Instruments, Inc., Milano, Italy) and XE-100 (PSIA, Inc., Santa Clara, CA, USA), operating in ambient air and a nitrogen atmosphere. A hygrometer (Tecpel co., Ltd., Taipei, Taiwan) was used to measure humidity and temperature. Adhesion and lateral force measurements in ambient air were mainly performed with PSIA equipment. The lateral force and topography of organic molecule patterns in air were imaged in contact mode using silicon AFM probes (NSC36; PSIA, Inc., Santa Clara, CA, USA) with soft cantilevers (nominal spring constant of 0.6 N/m) and sharpened conical tips (nominal curvature radius of 10 nm).

The experiments in environmental controlled conditions such as constant humidity and nitrogen atmosphere were performed with the Nanoscope IIIa. The adhesion and lateral forces of organic molecule monolayers were measured in contact mode using triangular silicon nitride cantilevers (NPS, 15 nm radius) with a nominal force constant of 0.58 N/m (manufacturer’s specification). The gold ball tip was used to increase the surface area to be modified by peptides for measuring mixed organic molecule monolayers. Its scanning mode was contact mode and the gold ball on cantilevers (nominal force constant of 0.06 N/m) was 1 μm in diameter. All images were acquired at a line scan rate of 0.5 Hz.

## 3. Results and Discussion

Specific interactions in the aqueous phase are based on multiple hydrogen bonds and electrostatic attractions; however, van der Waals interactions are dominant between chemicals in the gas phase. To analyze the delicate affinity between molecules, two modes of scanning were evaluated in parallel: force-distance curve and lateral force mode.

### 3.1. Force-Distance Curve for SAMs of Various Chemicals

**Figure 1:**
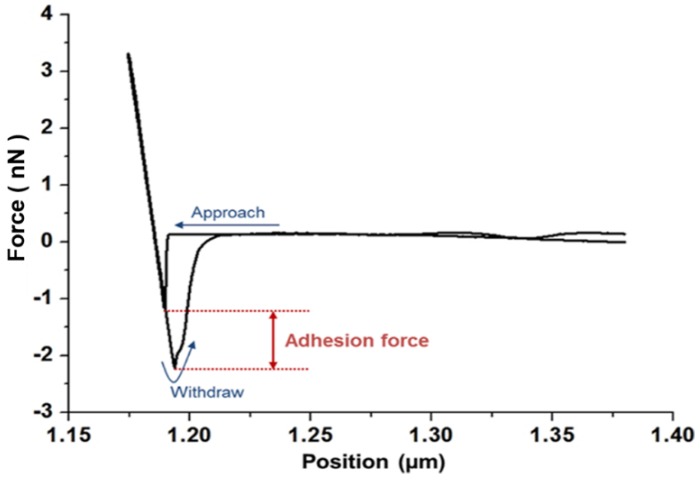
Force-distance curve of UDT.

For most biomolecules, the adhesion force was measured through force-distance (F-D) curves. As shown in Figure 1, the adhesion force was calculated from the retraction curve. The distribution of the adhesion force on the MCH layer was measured 100 times using the same silicon tip. The most probable adhesion force was determined by Gaussian fitting of the histogram for the distribution of force. In order to measure the tip-to-tip variation of adhesion force, several individual tips were compared. Figure S4 (Supplementary Information) shows the variation in the adhesion forces to MCH SAM within the silicon tips. The results from five tips showed very similar values within about 10% standard deviation (STD). The variation most likely originated from the inherent variability in cantilever characteristics and laser alignment for each measurement.

One major and arresting aspect of measurements performed in the gas phase is the formation of a water meniscus resulting from condensation of water between two objects. Thus, force measurements should be performed under controlled humidity; optimal conditions are dependent on the hydrophobicity of the substrates [18,25]. The effect of humidity on force measurements was stable around 50% relative humidity (RH) in Figure S5 (Supplementary Information). This result is in agreement with a report that long range force disappears over 50% RH [26].

When we compared the affinity of silicon tips to the different chemical groups listed in Table 1, UDT showed a weak affinity due to its hydrophobicity. Natural oxide layers are hydrophilic; therefore, tips covered with a natural oxide layer adhere more strongly to MCH and MUA with alcohol and carboxyl groups, respectively. However, it was hard to differentiate between MCH and MUA with respect to which molecule had a stronger interaction with the silanol group on the AFM tip.

**Table 1:** Adhesion force and contact angle between silicon tips and different chemical groups; alcohol (MCH), carboxyl (MUA) and alkyl (UDT) groups.

Functional Group	Contact Angle (°)	Adhesion Force (nN)
MCH	40	5.5 ± 0.38
MUA	20	5.6 ± 0.98
UDT	90	2.65 ± 0.31

### 3.2. Lateral Force for SAM of Various Chemicals

Lateral force measurements can be an alternative method for analyzing chemical interactions. The lateral deflection of the cantilever is recorded as the sample is scanned perpendicular to the cantilever axis. Precision of lateral force measurements was evaluated in a similar way to the F-D curves. Tip to tip variation in lateral force measurements was minor according to repeated experiments on substrates with the same chemical pattern (Figure S6). The standard deviation of the differential lateral force was 9%, which was similar to the F-D curve’s 10%. This mode is more advantageous in comparing the interaction forces relatively through the contrast of lateral force images.

MUA with a carboxyl group was printed on clean gold surface utilizing microcontact printing (μCP). The protocol for utilizing μCP is shown in Figure S2 in parallel with images of the fabricated stamps. The bare gold area was coated with UDT or MCH, resulting in two kinds of SAMs patterned on one substrate. The lateral force and topological images were acquired simultaneously in contact mode using a silicon tip with a loading force of 3 nN under ambient conditions.

**Figure 2:**
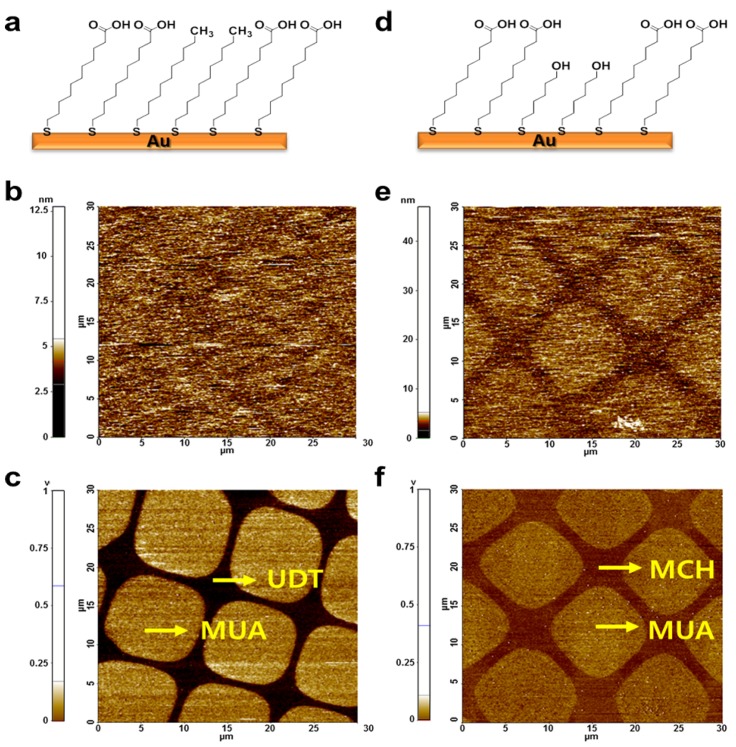
Chemical structures of MUA/UDT (**a**) and MUA/MCH; (**b**) and MUA/MCH; (**c**) and MUA/MCH; (**d**) the topography image of MUA/UDT; (**e**) the lateral force image of MUA/UDT; (**f**) using a silicon tip.

Figure 2 shows the topological and lateral force images of MUA/UDT and MUA/MCH patterned substrates. MUA and UDT with similar carbon chain lengths exhibited little difference in topography, but the contrast between lateral force images was strong due to their hydrophilic and hydrophobic character. Topological images of similar hydrophilic layers, MUA and MCH, showed a difference of 0.6 nm in the length of their carbon chains. According to Figure 2f, MUA had a stronger interaction (brighter = stronger attraction) with the silicon tip than MCH, indicating that lateral force images discriminated chemical groups more precisely. The dominant effect of hydrophilicity is assumed to render the image more clear-cut with with vague topological contrast.

We also carried out measurements under different humidity levels. When the humidity was under 50% the lateral force fluctuated much less in line with the effect of humidity (Figure S5). The lateral force on hydrophobic SAM increased slowly up to 60% RH and rapidly increased thereafter. However, the lateral force on hydrophilic substrates decreased slowly and consistently with humidity. Thus, all of the lateral force images were achieved under 50% RH.

### 3.3. Lateral Force Imaging for Simple Chemical Interactions

Imaging is very effective in recognizing lateral changes. To compare the relative lateral forces on multiple chemicals, we used sequential µCP of multiple chemical patterns partially overlapping each other. First, one of the organic molecules is printed in one shape, and then, the other is subsequently printed in another shape.

We utilized two stamps that were ODT printed in a square pattern 5 µm in width and MUA printed in a line pattern 4 µm in width. Through multiple chemical printing, a very flat substrate was produced with areas of individual chemicals. Since the SAMs of two chemicals coexisted within 100 µm, a single scan gave information on both octadecyl and carboxyl groups interacting with AFM tips. Thus, we avoided error from tip-to-tip variation and conditions for individual measurements.

**Figure 3:**
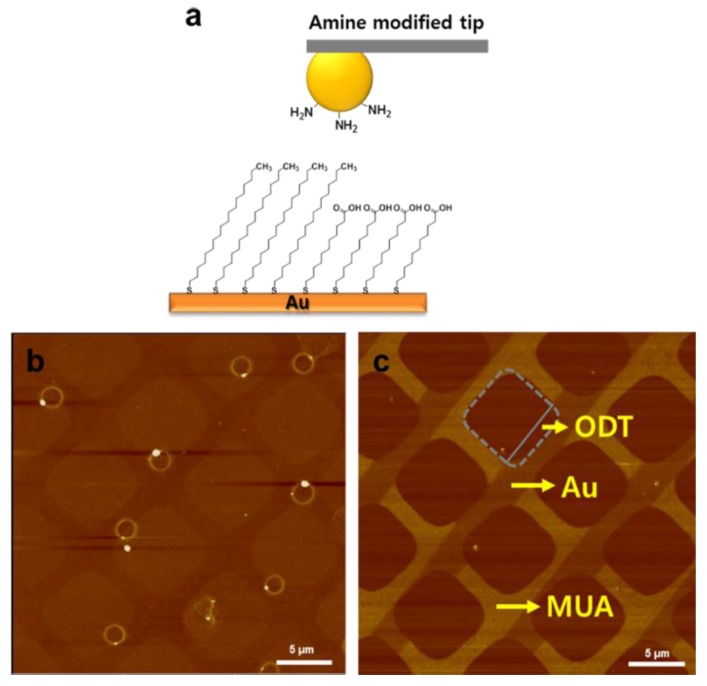
Chemical structure of the MUA/ODT pattern (**a**) Topography image (**b**) and Lateral force image (**c**) on the MUA/ODT pattern measured with an amine-modified ball tip (gray dash square: ODT pattern shape).

The interaction of amine group with ODT and MUA was selected as a model for lateral imaging of CFM. By utilizing ball tips, not conical tips, for amine-terminated layer, uniform and reliable SAM was completed on the side of CFM tip. According to the topological scans, the molecule heights were ODT > MUA > bare Au as shown in the Figure 3b. On the contrary, lateral forces were MUA > bare Au > ODT. As shown in Figure 3c, the line pattern (MUA) was much brighter than that of the square pattern (ODT), reflecting a higher affinity of MUA to the amine-functionalized tip. To interpret the lateral force images, we measured the water contact angle of each SAM and bare Au film; θ*_AUT_*, 40; θ*_MUA_*, 20; θ*_ODT_*, 100; and θ*_AU_*, 70. The stronger affinity of MAU/AUT might represent closer hydrophilicity between the pair of chemicals. However, the much brighter image for MUA compared to bare Au surface could not be explained only through hydrophilicity since the contact angles differ only by 20 (θ*_AUT_* — θ*_MUA_*) and 30 (θ*_AU_* — θ*_AUT_*), respectively. We think that the chemical affinity between amine and carboxyl groups might have added strength to the interaction of the AUT/MUA pair and, therefore, resulted in a distinct lateral image. It can be inferred that the chemically modified tip is advantageous to visualize chemical micropatterns than typical AFM tips while more convincing analysis could be achieved with chemicals of similar hydrophilicity on the same substrate.

### 3.4. Chemical Force Imaging with Various Peptides

Complex interactions composed of a set of electrostatic effects, dispersion forces and hydrogen bonds can be measured with CFM under aqueous conditions. If the interactions reside within a measurable force range, CFM might analyze such complicated ligand-receptor interactions in gas phase too. We compared the relative force between benzene and a couple of peptides that possess different affinity to phenyl group. Benzene is an important toxic gas in environments and one of the signature chemicals in breath [27,28]. Thus, we selected a benzene-specific peptide and a random peptide from a phage library. Each peptide was immobilized on individual tip through gold-thiol bonding. The target molecules were patterned using µCP with 1-octadecanethiol (ODT)/phenyl-terminated alkanethiol (PTA), which have methyl and phenyl groups, respectively. One stamp was printed with PTA in a line pattern 4 µm in width and the other was printed with ODT in a circle pattern 6 µm in width. 

**Figure 4:**
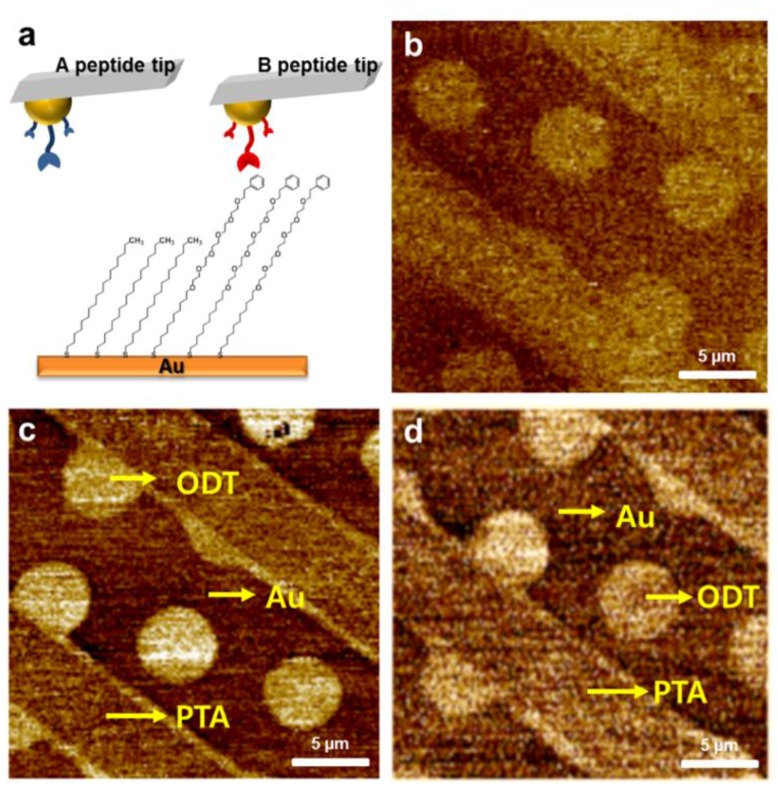
Chemical structure of the PTA/ODT pattern (**a**) Topography image and (**b**) Lateral force image of the PTA/ODT pattern measured using a tip modified with a random peptide (**c**) and benzene-specific peptide (**d**) benzene-specific peptide sequence: N terminus-AAGDMMAAPDP AC-C terminus and random peptide sequence: N terminus-AVPSGQAEADPAC-C terminus.

Figure 4 shows the topography and lateral force images on ODT/PTA. The heights of the molecules followed the order of ODT = PTA (2 nm) > bare Au according to the topological scan in Figure 4b. Lateral forces on each chemical zone were ranked in the order ODT > PTA > bare Au both with a random peptide and with benzene-specific peptide consistently, which can be explained with the hydrophobic alanine and valine at the outer ends of the two peptides. However, the line pattern (PTA) in Figure 4d is slightly brighter than that shown in Figure 4c, reflecting an additional affinity of the benzene specific-peptide to PTA layer. Dimensionless values of lateral forces in PTA zone with random peptide and the specific peptide were 0.04 and 0.28 respectively, whereas the value for ODT zone was 1.83 and 2.22 respectively. It seems that the lateral force is more dominantly influenced from the interaction of terminal groups. However, considering the same hydrophobicity of the peptide layers, we can suggest that the relatively brighter PTA zone with the benzene-specific peptide tip reflects a specific interaction between the peptide and phenyl group. In Table 2 below, averaged reaction forces between peptide and each chemical (Au, PTA, ODT) in the printed surface are listed.

**Table 2:** Lateral forces in each functional groups with random peptide and the specific peptide.

Functional Group	VPSGQAEA	AGDMMAAP
Au	−0.97 ± 0.20	−0.97 ± 0.72
PTA	−0.04 ± 0.30	0.28 ± 0.64
ODT	1.83 ± 0.38	2.22 ± 0.51

## 4. Conclusions

In this study, we evaluated forces between chemicals on an AFM tip and functionalized molecular monolayers. Microcontact printing generated monolayers of different chemicals patterned on a flat substrate. Lateral force imaging was able to discriminate the chemical patterns with high contrast for mixed patterns of ODT/MUA and ODT/PTA by utilizing chemically modified tips. With amine-terminated ball tips, carboxyl patterns appeared obviously brighter than intact Au or ODT patterns, supporting the carboxyl-amine interaction.

With peptide-conjugated tips on the pattern of ODT/PTA, we determined the interaction force between the phenyl group and the benzene-specific peptide as well as obvious hydrophobic interactions. As a result, we could provide clues to the interaction between benzene and a benzene-specific peptide compared with the case of the random peptide. This result indicates for the first-time that complex chemical interactions in the gas phase could be compared relatively through CFM. This method can be used in evaluating the affinity of volatile molecules to receptors for specific gas sensors.

## Supplementary Files

Supplementary File 1
