# Supplementary material for: Evaluation of Chemical Interactions between Small Molecules in the Gas Phase Using Chemical Force Microscopy
Source: Sensors (Basel). 2015 Dec 4;15(12):30683–92. doi: 10.3390/s151229823 (PMC4721743; doi:10.3390/s151229823)
Supplement: Supplementary File 1 [file sensors-15-29823-s001.pdf]

Supplementary Materials: Evaluation of Chemical Interactions between Small Molecules in Gas Phase Using Chemical Force Microscopy

Jieun Lee [1,2], Soomi Ju [1], In Tae Kim [1], Sun-Hwa Jung [3], Sun-Joon Min [3], Chulki Kim [4], Sang Jun Sim [2] and Sang Kyung Kim [1,*]

As shown in Figure S2, alkanethiol (**7**) containing a phenyl group at the terminal position was prepared in a short reaction sequence. Initially, treatment of (3-chloropropyl) benzene (**1**) with pentaethylene glycol produced the primary alcohol (**2**), which was subjected to etherification with 11-bromo-1-undecyl tert-butyldimethylsilyl ether to give silyl ether (**3**). Removal of the TBS protecting group of **3** with tetrabutylammonium fluoride provided alcohol (**4**), which was subsequently transformed to the corresponding mesylate (**5**) in high yield. Finally, we obtained the desired alkanethiol (**7**) by a displacement reaction of (**5**) with potassium thioacetate followed by acid-promoted hydrolysis.

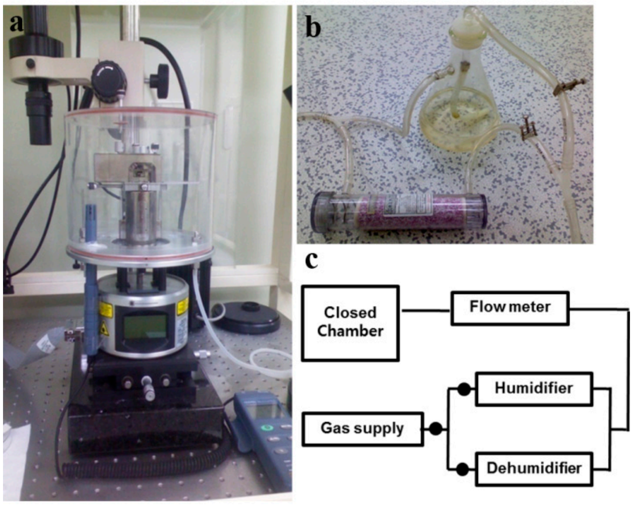

Figure S1. (**a**) Environmental hood and (**b**) humidifier equipment to control relative humidity & inert gas (**c**) Conceptual diagram of humidity and gas control system.

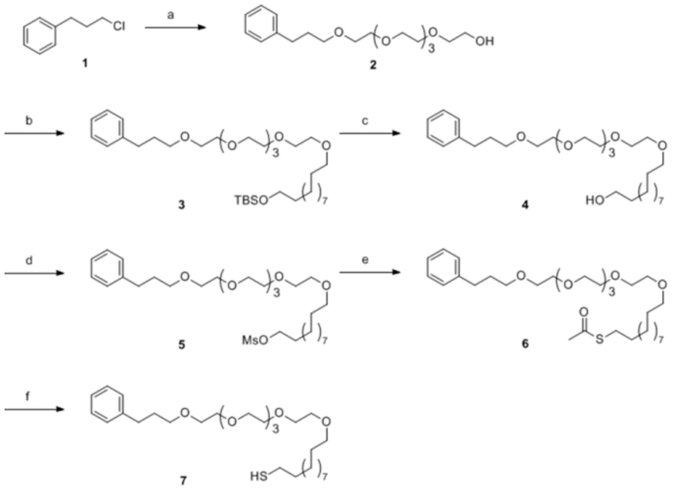

Figure S2. Synthesis of phenyl terminated alkanethiol (PTA) **7**. *Reagents and Conditions*: (**a**) pentaethylene glycol, NaH, KI, DMF, 0 °C to rt, 65%; (**b**) TBSO(CH2)11Br, NaH, DMF, 0 °C to rt , 47%; (**c**) TBAF, THF, rt, 97%; (**d**) MsCl, Et3N, CH2Cl2, rt, 92%; (**e**) CH_3COSK, DMF, rt, 79%; (**f**) HCl, MeOH, 60 °C, 96%.

Effect of relative humidity

Humidity is an important factor that can influence measurements of interaction force. MCH and UDT are known to form stable and densely packed two-dimensional crystal structures on a substrate of Au(111). Interaction between hydrophilic silicon nitride tips and the surface of hydrophilic MCH or hydrophobic UDT was investigated according to varying levels of relative humidity in an environmentally-controlled chamber. To minimize signal drift, the AFM and sample were equilibrated together for 1 h. Figure S3-A shows the adhesion force of the silica tip to hydrophilic and hydrophobic layers under widely varied relative humidity. The force on hydrophilic and hydrophobic SAM increased with humidity. According to the increase in humidity, a larger meniscus formed and increased the force between the tip and hydrophilic or hydrophobic surfaces. The humidity effect was relatively bigger on the hydrophilic surface. The adhesion force on MCH increased about 50% when the humidity increased from 10 to 80%. The smaller force change on hydrophobic SAM, a 30% increase, suggests that relative humidity had a weak effect due to the slow condensation of water.

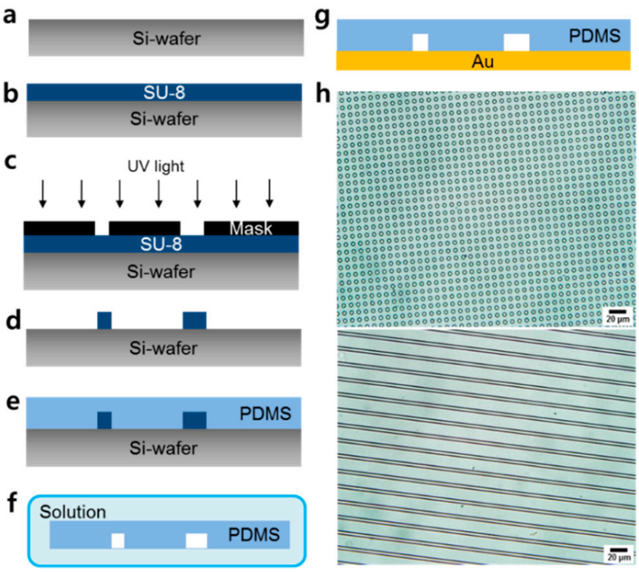

Figure S3. Simplified fabrication process of the SU-8 master mold and replication process for the PDMS stamp, (**a**) wafer cleaning, (**b**) SU-8 spin coating and soft baking, (**c**) UV exposure, (**d**) developing and post exposure baking, (**e**) PDMS replication, (**f**) soaking stamp, (**g**) stamping on gold substrate, (**h**) fabricated PDMS stamps (up: circles, down: lines).

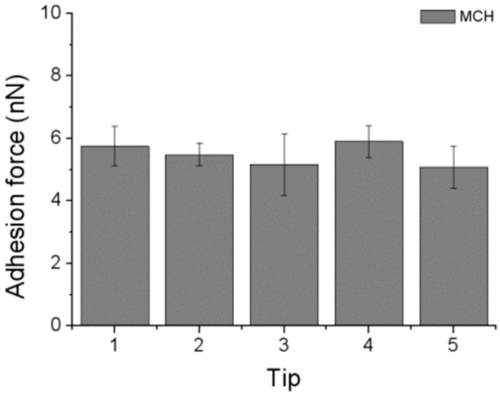

Figure S4. Tip-to-tip variation according to the adhesion force of MCH using a silicon tip.

Figure S5-B shows the lateral force change on hydrophilic and hydrophobic SAMs according to relative humidity. In this measurement, the load at the tip-sample contact was maintained at a constant force of 3nN. As the relative humidity increased, the lateral force decreased gradually and stabilized at 50% on a hydrophilic substrate. The decrease in lateral force indicates that the meniscus might act as a lubricant. In contrast, the lateral force on hydrophobic SAM slowly increased up to 60% and rapidly increased thereafter. The sudden increase in lateral force on UDT could have been caused by significant condensation of water molecules occurring at 70% RH. Above this humidity, special care has to be taken in lateral force analysis since the characteristics of the surface became dominated by a layer of water molecules.

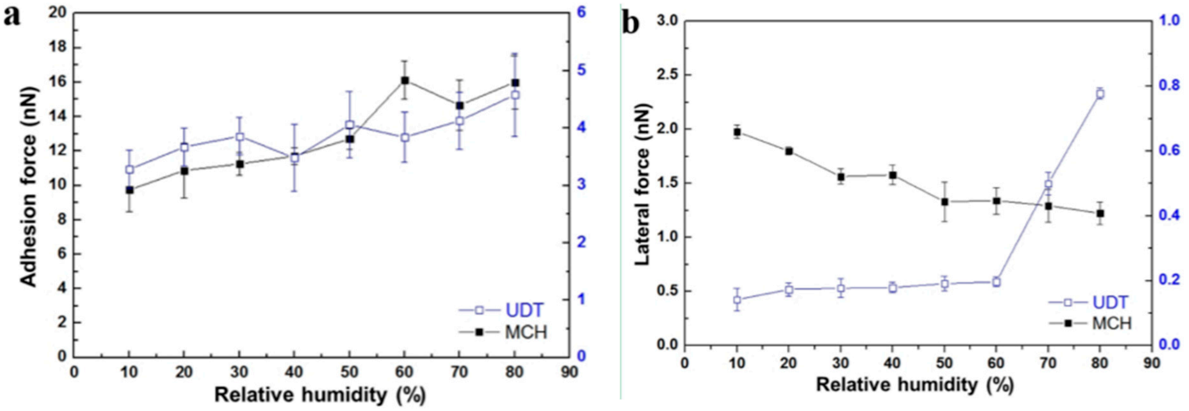

Figure S5. (**a**) Adhesion force change from the force distance curve of hydrophilic (MCH) and hydrophobic (UDT) SAMs according to varying levels of relative humidity. (**b**) Lateral force change of MCH and UDT according to relative humidity.

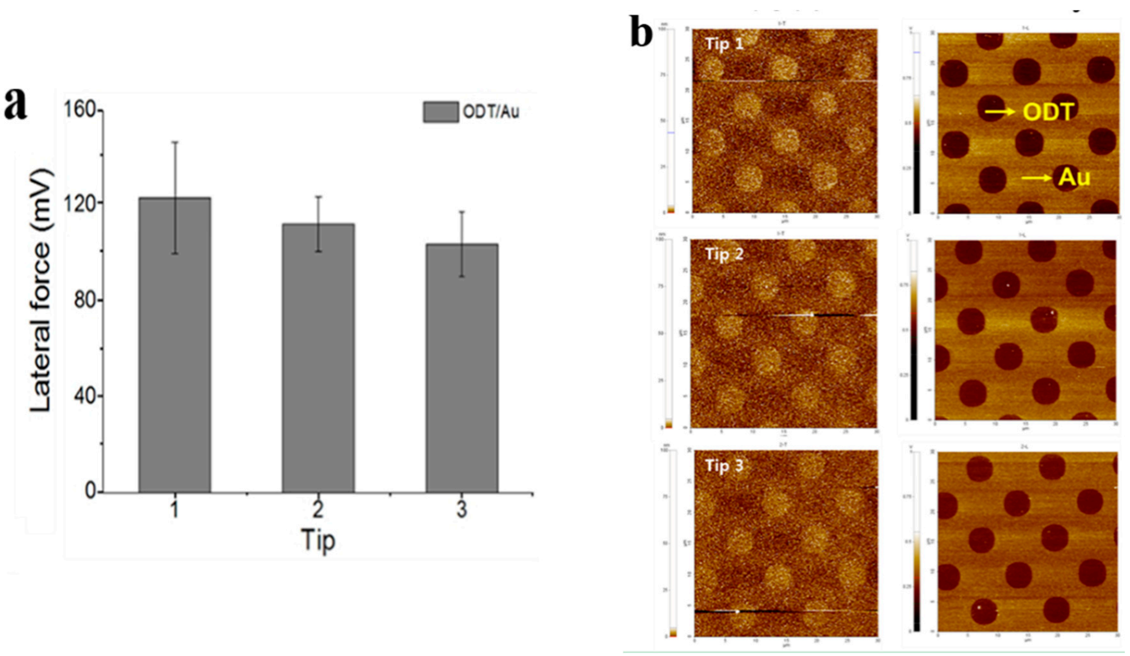

Figure S6. (**a**) Tip-to-tip variation according to the relative lateral force between Au and ODT using a ball tip. Chemical pattern of Au/ODT; (**b**) the topography image and lateral force image of Au/ODT using tip 1/tip2/tip3 in.
